# Prevalence of comorbidities in systemic sclerosis versus rheumatoid arthritis: a comparative, multicenter, matched-cohort study

**DOI:** 10.1186/s13075-018-1771-0

**Published:** 2018-12-04

**Authors:** Stylianos Panopoulos, Maria Tektonidou, Alexandros A. Drosos, Stamatis-Nick Liossis, Theodoros Dimitroulas, Alexandros Garyfallos, Lazaros Sakkas, Dimitrios Boumpas, Paraskevi V. Voulgari, Dimitrios Daoussis, Konstantinos Thomas, Georgios Georgiopoulos, Georgios Vosvotekas, Dimitrios Vassilopoulos, Petros P. Sfikakis

**Affiliations:** 1 Joint Rheumatology Program, 1st Department of Propedeutic Internal Medicine-Rheumatology Unit, National and Kapodistrian University of Athens, School of Medicine, Laikon General Hospital, Athens, Greece; 2 Rheumatology Clinic, Department of Internal Medicine, Medical School University of Ioannina, Ioannina, Greece; 3 Division of Rheumatology, University of Patras Medical School, Patras University Hospital, Patras, Greece; 4 4th Department of Medicine, Aristotle University, Thessaloniki, Greece; 5 Department of Rheumatology and Clinical Immunology, School of Health Sciences, University of Thessaly, Larissa, Greece; 6 Joint Rheumatology Program, 4th Department of Medicine, National and Kapodistrian University of Athens, School of Medicine, Attikon University Hospital, Athens, Greece; 7 Joint Rheumatology Program, Clinical Immunology-Rheumatology Unit, 2nd Department of Medicine and Laboratory, National and Kapodistrian University of Athens, School of Medicine, Hippokration General Hospital, 114 Vass. Sophias Ave, 115 27 Athens, Greece; 8 1st Department of Medicine, Aristotle University of Thessaloniki, School of Medicine, University General Hospital of Thessaloniki AHEPA, Thessaloniki, Greece

**Keywords:** Rheumatoid arthritis, Systemic sclerosis, Comorbidities, Cardiovascular diseases, Depression, Neoplasms

## Abstract

**Background:**

Comorbidities are common in chronic systemic connective tissue diseases and are associated with adverse outcomes, increased morbidity and mortality. Although the prevalence of comorbidities has been well-studied in isolated diseases, comparative studies between different autoimmune diseases are limited. In this study, we compared the prevalence of common comorbidities between patients with systemic sclerosis (SSc) and patients with rheumatoid arthritis (RA).

**Methods:**

Between 2016 and 2017, 408 consecutive patients with SSc, aged 59 ± 13 years (87% women), were matched 1:1 for age and gender with 408 patients with RA; mean disease duration was 10 ± 8 and 9 ± 8 years, respectively. Rates of cardiovascular risk factors, coronary artery disease, stroke, chronic obstructive pulmonary disease (COPD), osteoporosis, neoplasms and depression were compared between the two cohorts.

**Results:**

The prevalence of dyslipidemia (18.4% vs 30.1%, *p* = 0.001) and diabetes mellitus (5.6% vs 11.8%, *p* = 0.007) and body mass index (*p* = 0.001) were lower in SSc compared to RA, while there was no difference in arterial hypertension or smoking. While there was a trend for lower prevalence of ischemic stroke in SSc than in RA (1.1% vs 3.2%, *p* = 0.085), coronary artery disease was comparable (2.7% vs 3.7%). No differences were found between patients with SSc and patients with RA in the prevalence of COPD (5.2% vs 3.7%), osteoporosis (24% vs 22%) or neoplasms overall (1.1% vs 1.7%); however lung cancer was the most prevalent cancer in SSc (7/17, 41%), whereas hematologic malignancies (7/19, 36%) and breast cancer (7/19, 36%) predominated in RA. Depression was more prevalent in SSc (22% vs 12%, *p* = 0.001), especially in diffuse SSc.

**Conclusions:**

Despite the prevalence of dyslipidemia and diabetes mellitus in SSc being almost half that in RA, the cardiovascular comorbidity burden appears to be similar in both. The overall prevalence of neoplasms is no higher in SSc than in RA, but SSc has a more negative impact on quality of life, as clearly, more SSc patients develop depression compared to patients with RA.

## Background

Connective tissue diseases comprise over 100 disorders that are characterized by chronic inflammation and impaired quality of life. During the last two decades major therapeutic advances have been accomplished in this field, however patients with such disorders are clinically complex and in addition to the disease burden itself, the interplay between the disease activity and associated conditions may lead to increased morbidity and mortality. These comorbid conditions can preexist or arise as a result of the disease itself, and the concomitant treatments and continuous vigilance for prompt diagnosis and treatment is warranted.

Systemic sclerosis (SSc) is a rare disease that significantly affects quality of life and is characterized with one of the highest mortality rates among the various autoimmune diseases with an estimated standardized mortality ratio between 2.5 and 4.0 [1, 2]. Despite the impact of the disease in survival and quality of life, data on the epidemiology and clinical expression of comorbidities in SSc so far are limited. Conversely the interactions between disease-related inflammatory processes and development of several comorbidities has been thoroughly investigated in rheumatoid arthritis (RA) [3]. This is particularly the case for cardiovascular disease (CVD) [4], where RA is considered to be an independent predictor of the development of CVD with comparable CV risk to diabetes mellitus (DM) [5, 6].

To date there are no comparative studies examining the prevalence of the most relevant comorbidities in SSc versus RA. Our objective was to compare the prevalence of comorbidities in two multicenter, matched-cohorts of patients with SSc and RA.

## Methods

### Study population

In the current study we included patients with SSc and RA from six academic Rheumatology Departments across Greece with substantial experience in the management of connective tissue diseases. Patients with SSc were recruited consecutively between 2016 and 2017 and were matched 1:1 for age and gender with RA patients, all meeting the 1980 American College of Rheumatology (ACR) criteria [7] and the 1987 ACR criteria [8], respectively. RA patients were derived from the working cohort of an ongoing, multicenter prospective RA study involving 2491 patients (from the RA Study Group of the Greek Rheumatology Society) [9]. Ethical approval was provided by the local institutional boards of participating centers and informed consent was provided by all patients before their inclusion in the study. In both cohorts, information about cumulative comorbidity conditions was collected from the medical history records of the patients.

The comorbidities evaluated were dyslipidemia, DM, arterial hypertension, smoking, coronary artery disease (CAD), stroke, chronic obstructive pulmonary disease, osteoporosis, neoplasms and depression. Dyslipidemia was defined by total cholesterol of 240 mg/dl and/or low-density lipoprotein of 130 mg/dl and/or triglycerides of 160 mg/dl and/or intake of lipid-lowering drugs. A diagnosis of DM was based on the requirement for antidiabetic drugs. Arterial hypertension was defined by systolic blood pressure > 140 mmHg and/or diastolic blood pressure > 90 mmHg on two measurements or when antihypertensive treatment was administered after the diagnosis of hypertension was established. Patients receiving antihypertensive agents in the context of vasodilatory therapy not fulfilling criteria for hypertension were excluded from the analysis. Smoking was categorized as current smoker, ex-smoker or never smoked. CAD was defined by the presence of at least one of the following: myocardial infarction or angina pectoris based on clinical diagnosis and ischemic changes in the electrocardiogram and/or specific changes in cardiac enzymes and/or typical findings on coronary angiography. Stroke was defined by the presence of ischemic or hemorrhagic stroke based on clinical manifestations and/or supported by an imaging study. Chronic obstructive pulmonary disease (COPD) was defined by the administration of therapy for chronic bronchitis or emphysema. Osteoporosis was defined by bone density 2.5 standard deviations below that of a young adult measured by dual-energy x-ray absorptiometry. Νeoplasia diagnosis was based on patients’ medical records according to pathologic tissue biopsy of the affected organ. The diagnosis of depression was based on antidepressant therapy administered after psychiatric evaluation.

### Statistical analysis

Values related to categorical variables are expressed as percentages. Continuous variables are expressed as means ± SD. Data were compared using the chi-square test for categorical data and the Kruskal-Wallis test for continuous data. Association was tested using binomial logistic regression. Results are expressed as odds ratios (ORs). All analyses were carried out using the software Stata 13.1 and SPSS 21.

## Results

### Patient demographics

A total of 408 patients with SSc were included, 55% had the diffuse subtype, 89% were women, mean age at inclusion was 58.4 ± 14.5 years and patients were matched 1:1 for age and gender to 408 patients with RA. As shown in Table 1, disease duration was comparable between patients with SSc and patients with RA (10.1 ± 7.7 years vs 9.2 ± 7.8 years, respectively, *p* not significant (NS)), while no significant differences were observed in smoking or current treatment with corticosteroids (treatment duration and mean dose). On the other hand, body mass index (BMI) was significant higher in patients with RA (26.5 ± 4.86 vs 24.2 ± 3.61, *p* = 0.001). In Table 1, the characteristics of the limited and diffuse SSc subgroups and those of their matched patients with RA are also shown.

**Table 1:** Demographic characteristics of systemic sclerosis (SSc) and rheumatoid arthritis (RA) matched cohorts

	SSc	RA	Limited SSc	RA matched to limited SSc	Diffuse SSc	RA matched to diffuse SSc
Number of patients	408	408	182	182	226	226
Female, *n* (%)	364 (89%)	364 (89%)	169 (93)	169 (93)	195 (86)	195 (86)
Age at inclusion (years)	58.4 ± 13.5	57.5 ± 13.4	58.3 ± 12.6	57.4 ± 12.5	58.7 ± 14.2	57.9 ± 13.9
Disease duration (years)	10.1 ± 7.8	9.2 ± 7.7	8.85 ± 8.79	8.3 ± 7.39	10.7 ± 8.4	10.2 ± 7.9
Smoking, *n* (%)	104 (25.4%)	128 (31.3%)	54 (29.7)	59 (32.4)	50 (22.1)	69 (30.6)
Body mass index	24.2 ± 3.6	26.5 ± 4.9 ^*^	24.4 ± 4.0	26.8 ± 4.8 ^*^	24.1 ± 3.4	26.1 ± 4.7 ^*^
Current corticosteroid treatment	196 (48%)	185 (45%)	78 (43.1)	84 (46.7)	118 (48.7)	101 (44.7)
Duration (months)	14.6 ± 21.6	15.5 ± 26	14.1 ± 20.8	13.8 ± 36.5	15.2 ± 21.7	15.9 ± 36.9
Μean daily dose (mg)	2.8 ± 1.3	3.2 ± 1.5	2.3 ± 2.1	2.9 ± 3.2	3.1 ± 2.4	3.3 ± 2.6

Data are shown as mean ± 1 standard deviation

^*^p < 0.05

### Comorbidities

A comparative analysis of the prevalence of comorbidities between the two cohorts is shown in Table 2. Dyslipidemia (17.7% vs 30.2%, *p* = 0.001), and DM (5.6% vs 11.8%, *p* = 0.007) were less prevalent in SSc and RA, respectively, and the respective ORs were 0.5 (95% CI 0.36–0.69) and 0.45 (95% CI 0.27–0.75). On the contrary no differences were noted for the prevalence of arterial hypertension (31.8% vs 30.6%, respectively, *p* = 0.742). Despite the higher BMI and almost double the prevalence of dyslipidemia and DM in RA, no remarkable differences in coronary events (2.7% vs. 3.7%, *p* = 0.445; OR 0.73 (95% CI 0.33–1.60)) or stroke (1.9% vs 3.4%, *p* = 0.195; OR 0.56 (95% CI 0.23–1.35)) were noted between the two groups, although a trend for lower prevalence of ischemic stroke in patients with SSc than in patients with RA was observed (1.2% vs 2.9%, *p* = 0.085; OR 0.40 (95% CI 0.14–1.17)). Additionally, when we adjusted the analysis for corticosteroid treatment, hypertension and dyslipidemia, no relevant changes in the ORs were observed (Table 2).

**Table 2:** Prevalence of comorbidities in systemic sclerosis (SSc) and rheumatoid arthritis (RA) matched cohorts

Comorbidity	SSc	RA	Crude OR	Adjusted OR
Diabetes mellitus	23 (5.6)	48 (11.8)	0.45 (0.27–0.75)	–
Dyslipidemia	72 (17.7)	123 (30.2)	0.50 (0.36–0.69)	–
Arterial hypertension	131 (32.1)	125 (30.6)	1.07 (0.80–1.44)	–
Coronary event	11 (2.7)	15 (3.7)	0.73 (0.33–1.60)	0.74 (0.34–1.62)^*^
Stroke	8 (1.9)	14 (3.4)	0.56 (0.23–1.35)	0.55 (0.21–1.32)^*^
Ischemic stroke	5 (1.2)	12 (2.9)	0.40 (0.14–1.17)	0.39 (0.14–1.15)
Hemorrhagic stroke	3 (0.7)	2 (0.5)	1.5 (0.25–9.04)	1.48 (0.23–8.96)
Neoplasia	17 (4.2)	19 (4.7)	0.89 (0.46–1.74)	–
Chronic obstructive pulmonary disease	21 (5.2)	15 (3.7)	1.42 (0.72–2.80)	1.46 (0.74–2.90)^**^
Osteoporosis	98 (24.0)	92 (22.6)	1.09 (0.79–1.50)	1.08 (0.78–1.49)^***^
Depression	90 (22.1)	49 (12)	2.07 (1.42–3.03)	–

All data are shown as number (percentage)

*OR* odds ratio

^*^Adjusted for smoking, corticosteroid treatment, arterial hypertension, dyslipidemia

^**^Adjusted for smoking

^***^Adjusted for corticosteroid treatment

The overall prevalence of malignancies did not differ between SSc and RA (4.2% vs 4.7%, *p* = 0.733; OR 0.89 (95% CI 0.46–1.74)). However, there were differences in the type of neoplasms observed in the two cohorts. In particular, in the SSc cohort the most frequent type was lung cancer (7/17, 41%), while patients with RA developed mainly hematologic malignancies (7/19, 36%) and breast cancer (7/19, 36%).

Among the other comorbidities analyzed, the prevalence of chronic obstructive pulmonary disease (5.2% vs 3.7%, respectively, *p* = 0.326), osteoporosis or osteoporotic fractures was similar between the two groups (24% vs 22%, *p* = 0.619; OR 1.08 ([95% CI 0.78–1.49) and 6.1% vs 6.7%, *p* = 0.665; OR 1.13 (95% CI 0.64–2.0), respectively). However, depression (defined by the use of antidepressants) was almost twice as frequent in SSc compared to patients with RA (22% vs 12%, *p* = 0.001; OR 2.07 (95% CI 1.42–3.03), respectively).

In addition, sub-analysis between patients with limited (*n* = 182) or diffuse SSc (*n* = 226) and matched patients with RA was performed in order to examine the impact of SSc subtype on the prevalence of comorbidities. As shown in Table 3, with the exception of depression, no other differences were noted compared to the primary analysis. Dyslipidemia and DM were confirmed to be significantly less prevalent both in limited or diffuse SSc compared to RA, while no differences were noted in the prevalence of arterial hypertension, coronary events, stroke, malignancies, chronic obstructive pulmonary disease or osteoporosis. On the contrary, the sub-analysis revealed that depression was significantly more frequent in the diffuse SSc subgroup compared to matched patients with RA (27.2% vs 11.9%, *p* = 0.001; OR 2.753 (95% CI 1.675–4.523)), while it was comparable between patients with limited SSc and patients with RA (14.8% vs 12.1%, *p* = 0,455; OR 1.259 (CI 95% 0.688–2.304)).

**Table 3:** Prevalence of comorbidities in limited or diffuse systemic sclerosis (SSc) and rheumatoid arthritis (RA) matched controls

Comorbidity	Limited SSc *n* = 182	RA *n* = 182	OR	Diffuse SSc *n* = 226	RA *n* = 226	OR
Diabetes mellitus	12 (6.6)	23 (12.6)	0.485 (0.234–1.007)	11 (4.8)	25 (11.1)	0.408 (0.195–0850)
Dyslipidemia	34 (18.7)	56 (30.8)	0.513 (0.315–0.836)	38 (16.7)	67 (29.6)	0.475 (0.303–0.745)
Arterial hypertension	56 (30.8)	54 (29.7)	1.04 (0.668–1.635)	75 (33.2)	71 (31.4)	1.084 (0.731–1.609)
Coronary event	4 (2.2)	6 (3.3)	0.655 (0.182–2.363)	7 (3.1)	9 (4.0)	0.764 (0.279–2.087)
Stroke	5 (2.7)	6 (3.3)	0.824 (0.247–2.749)	3 (1.3)	8 (3.5)	0.363 (0.095–1.387)
Ischemic	3 (1.6)	5 (2.7)	0.590 (0.139–2.506)	2 (0.9)	7 (3.1)	0.277 (0.057–1.347)
Hemorrhagic	2 (1.1)	1 (0.5)	2.00 (0.18–22.252)	1 (0.4)	1 (0.4)	0.991 (0.062–15.944)
Neoplasia	7 (3.8)	8 (4.4)	0.865 (0.307–2.437)	10 (4.4)	11 (4.9)	0.897 (0.373–2.155)
Chronic obstructive pulmonary disease	8 (4.4)	6 (3.3)	1.341 (0.456–3.945)	13 (5.7)	9 (4.0)	1.758 (0.610–3.482)
Osteoporosis	41 (22.4)	39 (21.4)	1.09 (0.79–1.50)	57 (25)	52 (23)	1.115 (0.725–1.716)
Depression	27 (14.8)	22 (12.1)	1.259 (0.688–2.304)	63 (27.6)	27 (11.9)	2.814 (1.714–4.620)

## Discussion

It is well-established that comorbidities significantly contribute to the morbidity and mortality of rheumatic diseases; however, available data on their relative frequency and impact are limited [10–13]. Herein, we compared the prevalence of the most frequent comorbidities in two well-matched cohorts of patients with SSc and RA; to our knowledge this is the first such comparative study.

The most interesting result of the present study is that no significant differences in the occurrence of cardiovascular (CV) events were noted between patients with SSc and patients with RA, despite the fact that the prevalence of dyslipidemia and diabetes mellitus, two major risk factors for the development of CVD, was almost double in RA compared to SSc. Previous studies in patients with SSc have demonstrated an increased burden of CVD compared to the general population [14–16] despite a similar [17, 18] or even lower [15, 19] prevalence of traditional CV risk factors. It seems therefore that CVD in SSc is complex and multifactorial and except for atherosclerosis and chronic inflammation, other disease-specific mechanisms like microangiopathy and vasospasm may be key in the development of CVD in patients with SSc [20–25].

Microvasculopathy is well-documented in SSc and although not yet clearly elucidated through which mechanisms, it seems to contribute to the development of macrovascular disease, probably through pathologic processes like endothelial dysfunction, endothelial cell activation and impaired vascular remodeling. This hypothesis is supported by the reports from some studies that renal crisis [19] and pulmonary arterial hypertension (PAH) [15, 26], the two major clinical characteristics of microvasculopathy, are independent predictors of CVD. Moreover, the fact that PAH is in general more prevalent in SSc compared to RA [27] may reflect an increased burden of endothelial dysfunction in patients with SSc, enhancing the aforementioned hypothesis and potentially explaining the similar CV risk in the two diseases despite the lower prevalence of other common CVD risk factors in Patients with SSc. Finally, coronary vasospasm or myocardial Raynaud’s phenomenom, as it is usually called, contributes to a degree to the pathogenesis of CVD in SSc, most likely through the development of focal areas of myocardial fibrosis and contraction-band necrosis as a result of reperfusion injury [28].

Another interesting finding of our study was the higher prevalence of depression requiring treatment in patients suffering from diffuse SSc compared to RA. Several studies have investigated the prevalence of depression in rheumatic diseases with significant variability in the reported results, probably due to different criteria used to define depression. In a recent Canadian study of 345 patients the estimated depression rate in SSc was ~ 23% [29], while in a systematic review of eight studies the prevalence ranged from 36 to 65% [30]. In our study the respective rate based on the use of antidepressants was 22%.

On the other hand, in a meta-analysis of 72 studies in patients with RA, the prevalence of depression disorders ranged between 16.8% and 38.8% (depending on which assessment measures were applied) [31], while in our study it was somewhat lower (12%). The results of our study showed that SSc and especially the diffuse subtype exerts a greater psychosocial burden than RA, but these results are only indicative and properly conducted studies with standardized criteria for diagnosis of depression are needed in order to make secure conclusions.

There were no significant differences between the two groups in the other comorbidities that were analyzed. Of note, although the overall prevalence of neoplasms was comparable between the two groups, the most common neoplasm observed in patients with SSc was lung cancer, whereas patients with RA more often developed hematologic malignancies and breast cancer. The increased relative risk of development of lung cancer in SSc is well-established as shown in two meta-analyses [32, 33] and reflects the direct causal association between SSc and lung cancer due to the increased prevalence of interstitial lung disease and the presence of chronic lung inflammation. On the other hand, the increased prevalence of breast cancer in our RA cohort is in opposition to the results of other meta-analyses, which indicate reduced risk of breast cancer in RA [34, 35], but this discrepancy might be explained by the inevitable over representation of female gender in our RA cohort.

Our study has certain limitations that should be addressed. First, the RA cohort is not a random population sample and as mentioned previously is characterized by over representation of female patients, and the prevalence noted might be different from that in other RA populations. Another important limitation is the lack of information on the concomitant immunosuppressive therapy that patients had received and as a consequence the effect of such treatments in the development of various comorbidities was not analyzed. Also we did not use any standardized questionnaire to measure depression and the diagnosis of depression was based only on the administration of antidepressant treatment after psychiatric evaluation.

## Conclusions

To conclude, this is the first study in the literature directly assessing the prevalence of common comorbidities in SSc versus RA. These results indicate that SSc has comparable cardiovascular risk to RA but poses a greater negative impact on quality of life, as more patients with SSc develop depression. Further studies that will evaluate the epidemiology and pathophysiology of various comorbidities in different rheumatic diseases will provide additional information on the disease-specific risk factors and underlying mechanisms so as to define optimal preventive and therapeutic strategies.

## References

[1] Mok CC, Kwok CL, Ho LY, Chan PT, Yip SF (2011). Life expectancy, standardized mortality ratios, and causes of death in six rheumatic diseases in Hong Kong, China. Arthritis Rheum.

[2] Toledano E, Candelas G, Rosales Z, Martinez PC, Leon L, Abasolo L (2012). A meta-analysis of mortality in rheumatic diseases. Reumatol Clin.

[3] Dougados M, Soubrier M, Antunez A, Balint P, Balsa A, Buch MH (2014). Prevalence of comorbidities in rheumatoid arthritis and evaluation of their monitoring: results of an international, cross-sectional study (COMORA). Ann Rheum Dis.

[4] Kitas GD, Gabriel SE (2011). Cardiovascular disease in rheumatoid arthritis: state of the art and future perspectives. Ann Rheum Dis.

[5] Peters MJ, van Halm VP, Voskuyl AE, Smulders YM, Boers M, Lems WF (2009). Does rheumatoid arthritis equal diabetes mellitus as an independent risk factor for cardiovascular disease? A prospective study. Arthritis Rheum.

[6] Lindhardsen J, Ahlehoff O, Gislason GH, Madsen OR, Olesen JB, Torp-Pedersen C (2011). The risk of myocardial infarction in rheumatoid arthritis and diabetes mellitus: a Danish nationwide cohort study. Ann Rheum Dis.

[7] Preliminary criteria for the classification of systemic sclerosis (scleroderma). Subcommittee for scleroderma criteria of the American Rheumatism Association Diagnostic and Therapeutic Criteria Committee. Arthritis Rheum 1980;23:581–590. 10.1002/art.1780230510 7378088

[8] Arnett FC, Edworthy SM, Bloch DA, McShane DJ, Fries JF, Cooper NS (1988). The American Rheumatism Association 1987 revised criteria for the classification of rheumatoid arthritis. Arthritis Rheum.

[9] Thomas K, Lazarini A, Kaltsonoudis E, Drosos AA, Papalopoulos I, Sidiropoulos P (2018). Multicenter cross-sectional study of patients with rheumatoid arthritis in Greece: results from a cohort of 2.491 patients. Mediterr J Rheumatol.

[10] Rua-Figueroa I, Fernandez CM, Andreu JL, Sanchez-Piedra C, Martinez-Taboada V, Olive A (2017). Comorbidities in patients with primary Sjogren's syndrome and systemic lupus erythematosus: a comparative registries-based study. Arthritis Care Res (Hoboken).

[11] Nas K, Karkucak M, Durmus B, Karatay S, Capkin E, Kaya A (2015). Comorbidities in patients with psoriatic arthritis: a comparison with rheumatoid arthritis and psoriasis. Int J Rheum Dis.

[12] Wolfe F, Michaud K, Li T, Katz RS (2010). Chronic conditions and health problems in rheumatic diseases: comparisons with rheumatoid arthritis, noninflammatory rheumatic disorders, systemic lupus erythematosus, and fibromyalgia. J Rheumatol.

[13] McCarey D, Sturrock RD (2009). Comparison of cardiovascular risk in ankylosing spondylitis and rheumatoid arthritis. Clin Exp Rheumatol.

[14] Avina-Zubieta JA, Man A, Yurkovich M, Huang K, Sayre EC, Choi HK (2016). Early cardiovascular disease after the diagnosis of systemic sclerosis. Am J Med.

[15] Ngian GS, Sahhar J, Proudman SM, Stevens W, Wicks IP, Van DS (2012). Prevalence of coronary heart disease and cardiovascular risk factors in a national cross-sectional cohort study of systemic sclerosis. Ann Rheum Dis.

[16] Ngian GS, Sahhar J, Wicks IP, Van DS (2011). Cardiovascular disease in systemic sclerosis–an emerging association?. Arthritis Res Ther.

[17] Man A, Zhu Y, Zhang Y, Dubreuil M, Rho YH, Peloquin C (2013). The risk of cardiovascular disease in systemic sclerosis: a population-based cohort study. Ann Rheum Dis.

[18] Khurma V, Meyer C, Park GS, McMahon M, Lin J, Singh RR (2008). A pilot study of subclinical coronary atherosclerosis in systemic sclerosis: coronary artery calcification in cases and controls. Arthritis Rheum.

[19] Nordin A, Jensen-Urstad K, Bjornadal L, Pettersson S, Larsson A, Svenungsson E (2013). Ischemic arterial events and atherosclerosis in patients with systemic sclerosis: a population-based case-control study. Arthritis Res Ther.

[20] Au K, Singh MK, Bodukam V, Bae S, Maranian P, Ogawa R (2011). Atherosclerosis in systemic sclerosis: a systematic review and meta-analysis. Arthritis Rheum.

[21] D'Angelo WA, Fries JF, Masi AT, Shulman LE (1969). Pathologic observations in systemic sclerosis (scleroderma). A study of fifty-eight autopsy cases and fifty-eight matched controls. Am J Med.

[22] Tarek e-G, Yasser AE, Gheita T (2006). Coronary angiographic findings in asymptomatic systemic sclerosis. Clin Rheumatol.

[23] Bartoli F, Blagojevic J, Bacci M, Fiori G, Tempestini A, Conforti ML (2007). Flow-mediated vasodilation and carotid intima-media thickness in systemic sclerosis. Ann N Y Acad Sci.

[24] Derk CT, Jimenez SA (2007). Acute myocardial infarction in systemic sclerosis patients: a case series. Clin Rheumatol.

[25] Bulkley BH, Klacsmann PG, Hutchins GM (1978). Angina pectoris, myocardial infarction and sudden cardiac death with normal coronary arteries: a clinicopathologic study of 9 patients with progressive systemic sclerosis. Am Heart J.

[26] Komocsi A, Pinter T, Faludi R, Magyari B, Bozo J, Kumanovics G (2010). Overlap of coronary disease and pulmonary arterial hypertension in systemic sclerosis. Ann Rheum Dis.

[27] Ahmed S, Palevsky HI (2014). Pulmonary arterial hypertension related to connective tissue disease: a review. Rheum Dis Clin North Am.

[28] Kahaleh B (2008). Vascular disease in scleroderma: mechanisms of vascular injury. Rheum Dis Clin N Am.

[29] Jewett LR, Razykov I, Hudson M, Baron M, Thombs BD (2013). Prevalence of current, 12-month and lifetime major depressive disorder among patients with systemic sclerosis. Rheumatology (Oxford).

[30] Thombs BD, Taillefer SS, Hudson M, Baron M (2007). Depression in patients with systemic sclerosis: a systematic review of the evidence. Arthritis Rheum.

[31] Matcham F, Rayner L, Steer S, Hotopf M (2013). The prevalence of depression in rheumatoid arthritis: a systematic review and meta-analysis. Rheumatology (Oxford).

[32] Bonifazi M, Tramacere I, Pomponio G, Gabrielli B, Avvedimento EV, La VC (2013). Systemic sclerosis (scleroderma) and cancer risk: systematic review and meta-analysis of observational studies. Rheumatology (Oxford).

[33] Onishi A, Sugiyama D, Kumagai S, Morinobu A (2013). Cancer incidence in systemic sclerosis: meta-analysis of population-based cohort studies. Arthritis Rheum.

[34] Smitten AL, Simon TA, Hochberg MC, Suissa S (2008). A meta-analysis of the incidence of malignancy in adult patients with rheumatoid arthritis. Arthritis Res Ther.

[35] Simon TA, Thompson A, Gandhi KK, Hochberg MC, Suissa S (2015). Incidence of malignancy in adult patients with rheumatoid arthritis: a meta-analysis. Arthritis Res Ther.

